# KNODWAT: A scientific framework application for testing knowledge discovery methods for the biomedical domain

**DOI:** 10.1186/1471-2105-14-191

**Published:** 2013-06-13

**Authors:** Andreas Holzinger, Mario Zupan

**Affiliations:** 1 Research Unit Human-Computer Interaction (HCI4MED), Institute for Medical Informatics, Statistics and Documentation, Medical University Graz, Auenbruggerplatz 2/V, Graz 8036, Austria; 2 Institute for Genomics and Bioinformatics, Graz University of Technology, Petersgasse 14, Graz 8010, Austria; 3 Institute for Information Systems and Computer Media, Graz University of Technology, Inffeldgasse 16c, Graz 8010, Austria

**Keywords:** Knowledge discovery, Methods, Data analytics

## Abstract

**Background:**

Professionals in the biomedical domain are confronted with an increasing mass of data. Developing methods to assist professional end users in the field of Knowledge Discovery to identify, extract, visualize and understand useful information from these huge amounts of data is a huge challenge. However, there are so many diverse methods and methodologies available, that for biomedical researchers who are inexperienced in the use of even relatively popular knowledge discovery methods, it can be very difficult to select the most appropriate method for their particular research problem.

**Results:**

A web application, called KNODWAT (KNOwledge Discovery With Advanced Techniques) has been developed, using Java on Spring framework 3.1. and following a user-centered approach. The software runs on Java 1.6 and above and requires a web server such as Apache Tomcat and a database server such as the MySQL Server. For frontend functionality and styling, Twitter Bootstrap was used as well as jQuery for interactive user interface operations.

**Conclusions:**

The framework presented is user-centric, highly extensible and flexible. Since it enables methods for testing using existing data to assess suitability and performance, it is especially suitable for inexperienced biomedical researchers, new to the field of knowledge discovery and data mining. For testing purposes two algorithms, CART and C4.5 were implemented using the WEKA data mining framework.

## Background

Professionals in the biomedical domain collect, process and analyze large amounts of data, generally referred to as Big Data. Data exploration has been hailed as the fourth paradigm in the investigation of nature, after empiricism, theory and computation [1]. The introduction to the 2011 International Conference on Bioinformatics [2], included some interesting, yet dramatic statements about the size of this Big Data, e.g. that genomic data is reaching tsunami proportions [3], while at the same time, its clinical applications are rather a slowly rising tide [4]. Moreover, the ability to perform complex experimental work on the computer, in addition to the laboratory, further increases freely distributed raw data on the Web [5].

For all these reasons, experts in Bioinformatics are confronted with increased volumes of highly complex and often weakly-structured data [6-8]. Research in Human-Computer Interaction (HCI) and Knowledge Discovery in Data and Data Mining (KDD), has long been working to develop methods that help end users to identify, extract, visualize and understand useful information from the huge amount of high dimensional [9] and often weakly structured and/or non-standardized data [10,11]. Supporting professional end users in understanding their data without becoming overwhelmed, while keeping the cognitive effort of the computational processes low [12], so that the experts may concentrate on their scientific work, is a great challenge. Various approaches, including statistical and graphical-theoretical methods, data mining, and computational pattern recognition, have been applied to this task in the past with varying success [13]. Meanwhile, there are so many diverse methods and methodologies available [14-17], each of these having strengths in some areas and weaknesses in other areas. Such knowledge discovery methods are used to find patterns, similarities, anomalies, relationships etc. and other relevant information inside of highly complex data sets with the aim of obtaining insight into the data and towards sensemaking [18,19].

Consequently, such methods can greatly increase the efficiency of research in bioinformatics [20-29]. One of the biggest problems faced by researchers who want to use such knowledge discovery methods in their daily practice is, that there is no overall best method for each data set following the Şno free lunch theoremŤ [17] and even an expert may not be able to recommend the application of a particular method to a particular problem without knowing details about the data.

Hence, finding out which of the available, well studied approaches is the best one for a certain data set is a difficult task. Depending on the size of the research project, the necessary effort to find a suitable method might be too great, especially if there is no efficient method to benchmark the used data on a large variety of different algorithms. In order to help researchers deal with the problem of finding a suitable method for knowledge discovery on their data, we have developed a software called KNODWAT (KNOwledge Discovery With Advanced Techniques), which is an extensible application framework for testing knowledge discovery methods [30]. The application provides features to manage projects and social features, and to administrate as well as end-users including sharing and commenting on data. Moreover, by adding new knowledge discovery methods, it can be easily extended in various areas, thereby enabling researchers to test their own data with diverse, intuitive methods and compare the results in order to select the most suitable method for their particular data set. It is not necessary to know or understand the functionality of the algorithms behind these methods. The focus during the planning and implementation of the framework was on keeping it generic and extensible enough for a wide audience, especially for novice researchers in bioinformatics; but also to provide a strong functional body and an intuitive user interface to make it accessible and useful for researchers without a lot of experience in the field of machine learning.

## Implementation

**Figure 1:**
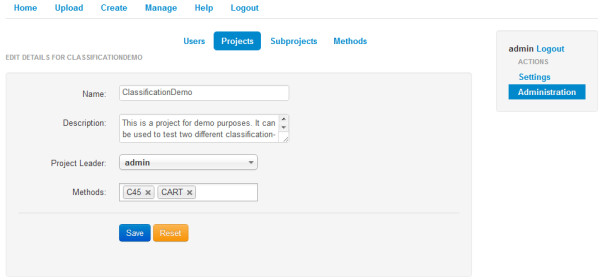
Overview screen inside the KNODWAT application.

The KNODWAT framework (see Figure 1) was implemented using the Java programming language based on the Spring framework 3.1. For data modeling and persistence, Hibernate framework 3.5.1 was used, mainly due to the very good Spring integration of the framework. The software runs on Java 1.6 and above and requires a web server such as Apache Tomcat and a database server such as the MySQL Server. For frontend functionality and styling, Twitter Bootstrap was used as well as jQuery for interactive user interface operations. The two already integrated algorithms, CART and C4.5 were implemented using the WEKA data mining framework [31-33].

Typical for web-based applications, the framework follows the Model View Controller software architecture pattern [34]. The general architecture behind the framework allows for the extension the core functionality using the service classes, tag libraries and utilities that are already available. The addition of new algorithms to the application was implemented using the strategy design pattern, encapsulating the different algorithms for the same task and making them interchangeable, which should help developers add new functional algorithms to the project without having to know the inside of the actual framework.

Arguably the most important architectural, or rather general design decision regarding the KNODWAT framework was to make it a web-based application. This crucial decision was based on three factors: usability, multi platform compatibility and the social aspect of research. With KNODWAT being aimed at researchers from all disciplines of science, especially at people with little experience in information technology [35], introducing new users to the framework will be easier with the user interface similar to widely used services such as Twitter and Youtube. Even with little IT knowledge and experience, there is a high chance, that a researcher has been using the web, including search engines and social networks to communicate and find resources. This assumption leads to the conclusion, that a web interface based on the general design principles of well known services should make it easier for inexperienced users to be introduced to the application. The second concern was multi platform availability for the application. Due to the popularity of both smartphones and tablets, there is an inherent need to make applications available to static and mobile devices [36]. This can be difficult, considering the different technologies involved in creating mobile applications (Android, iOS, Windows Mobile.), but all of these devices have web browsers installed, capable of displaying complex web applications and enabling user interaction on the same level as a PC. The third reason for making KNODWAT web-based was the social component of research meaning the creation and sharing of results with other people, or following other researchers’ progress.

### Extension

In order to make the KNODWAT framework applicable in many different disciplines of science, it has to be easy to add new methods to the existing platform. Currently it is not possible to extend the framework without any software engineering experience, but considering the way in which the extensibility functionality of KNODWAT is built, even a rather inexperienced programmer with some knowledge of the Java programming language and the ability to follow a few simple, well documented, steps is able to add new algorithms to the application. The whole extension process revolves around the concept of convention over configuration, using the Java reflection framework to wire the different components together automatically when they are named correctly and located in the right places within the project.

The first step is to create a new configuration object for the chosen algorithm. Basically, this object represents the custom parameters used for the algorithm. In the case of CART, one parameter will be created to enable the user to prune trees and another one to control how many data elements are used for training. It is important to note, that the extension of KNODWAT works by the use of convention over configuration, which means, that the naming of the created classes is relevant, as it will be used to link the created parts together. The configuration object is a *POJO*−*PlainOldJavaObject*, where the setter- and getter methods are annotated with specifications regarding their later use for the automatic wiring.

After the configuration object has been created, the next step is to create an implementation class, extending the *DefaultMethodImpl* class. This class has to override the *run*() method, where the execution of the algorithm will take place. The developer can freely create other methods, such as helper methods, but the *run*() method will always be called during method execution. The parameters as well as the input files are inside the *Map*<*String*,*Object*>*data*, which is the method parameter of the *run*() method. Inside this data map, all relevant input objects can be found, identifiable by their names. The handling of these input objects will differ from algorithm to algorithm and has to be implemented according to the individual needs of the method at hand. If anything goes wrong, for example an exception occurs, it is advisable to use the *fireErrorEvent*() method, in order to let users know that the method execution could not be performed successfully and what went wrong. Another method with regard to events is *fireStartEvent*, which can be used, for example, after all input parameters and data have been validated and the execution can start. The third event function, *fireSuccessEvent*, is called automatically when the result creation is triggered.

The execution of the algorithm can start after the validation of input data and necessary parameters. When the algorithm is completed, the created output data has to be saved. Once these result objects have been created, they are added to a list containing *GeneralResult* objects, describing a collection of output data. This output data list is then passed on to the method *createResult*(), which handles the persistence of a result object, adds and persists all the output data and fires the success event if everything worked, concluding the method execution. In essence, the developer responsible for adding a new method to the KNODWAT framework has to create a class, override a method, create a list containing output data within this method and call the *createResult*() method, which should be manageable for people with some experience in the Java programming language and guided by this tutorial.

Once both the configuration object and the implementation class have been created, positioned and named correctly, the only things left to do are to create a suitable view for the result detail and to create a database entry, which makes the method usable.

The last step is to create a custom JSP view file for presenting the results generated by the newly added algorithm. The developer should have some basic knowledge on how to create Java Server Pages, especially if the goal is to present the results in an attractive and neat way, providing a high amount of transparency and clarity. Nonetheless, the creation of this view is, again, fairly simple. All of the output data objects are available in the <*DOLLAR*/>{*outputData*} object and accessible via the JSTL. There are no limits regarding frontend “magic” such as JavaScript based animations or widgets, as the created view will be included within the existing *result*.*jsp* view and is not restricted in any way.

After completing these three simple steps; the newly added algorithm can be used throughout the framework. It is of course advisable to execute some intensive testing before releasing an extended version of the framework, as bugs and errors regarding the convention over configuration concept behind the extension feature can lead to instability within the whole application.

### Comparison to other Software in the field

The KNODWAT application framework was greatly inspired by existing software in the field, most notably Orange [37], for its extensibility and modular approach and WEKA [31] for its abundance of implemented and tested algorithms within the field of knowledge discovery. KNODWAT however, while generally providing similar features as the above mentioned software, as well as other projects such as the Weka Web Interface [38] or KNIME [39], differs from them in several aspects. One of these aspects is, that KNODWAT is a web-based platform built for user interaction and collaboration between researchers. It not only provides an interface for using existing algorithms, such as the Weka Web Interface, which is web-based as well, but also provides social features for organizing research projects and to enable sharing of data and results both within and outside of the platform. Another relevant aspect of KNODWAT, setting it apart from other software in the field is the fact that it was built with a focus on extensibility, meaning that the software is not meant to be a static library providing a certain amount of functionality, but rather an evolving platform which can be shaped according to the need of any user base. The KNODWAT user interface was specifically designed to make it easier for researchers with little experience in computer science and machine learning to use existing algorithms on their data, making these powerful knowledge discovery tools available to a broader audience of researchers, setting it apart from a non-technical perspective.

The main difference between KNODWAT and other knowledge discovery applications is that it is web-based. There are several advantages to this approach, the most important one being that most users, experienced with machine learning techniques or not, have used web applications such as social networks or search engines before and are thus more familiar with the general workings of them and the standard user interactions. This familiarity makes it easier to access the application, there is no need to download or install a bulky piece of software nor is there a need to regularly check for updates, users simply have to create an account and are ready to use the platform. Another advantage is compatibility, as web applications work on just about any device, which gets more and more important considering the increasing market share of mobile devices such as tablets. The use of web-based clustered computation services and social network applications, while also possible in standalone applications, is more intuitive with web-based applications. The main drawbacks of applications based on the world wide web are concerns regarding security and data privacy, which are of course relevant issues for many research projects. In general, social features for collaboration in the form of sharing data and the methods with which the results were found are easier to implement and more intuitive to use in an already connected environment such as the world wide web, and with the prime motivation behind the project - the spreading of awareness and increased accessibility of knowledge discovery techniques implementing KNODWAT as a web-based application seemed obvious.

## Results

This section presents a feature list of the completed KNODWAT framework as well as a small knowledge discovery study using two biomedical data sets, which was conducted using KNODWAT.

### Features

The KNODWAT (Knowledge Discovery With Advanced Techniques) is an extensible application framework for testing knowledge discovery methods. The current version, provides many features, the most important ones being the following: 

• Web-based user interface designed for easy accessibility

• Localization for English and German

• High performance result creation

• Multi-file upload system

• Helpful documentation for beginners

• Event based notification system

• Dynamic content filtering methods for fast navigation

•Simple content management

• Easy extension capabilities for knowledge discovery algorithms

• Multiple user accounts and roles

• Full administration capabilities within the system

• Social features such as following, sharing and commenting

• Multi-platform compatibility

### Usage

The basic KNODWAT application supports three different user roles: 

• Administrator (no restrictions)

• Researcher (result creation, project administration, data upload, view own and shared content)

• User (can view and comment on shared content)

**Figure 2:**
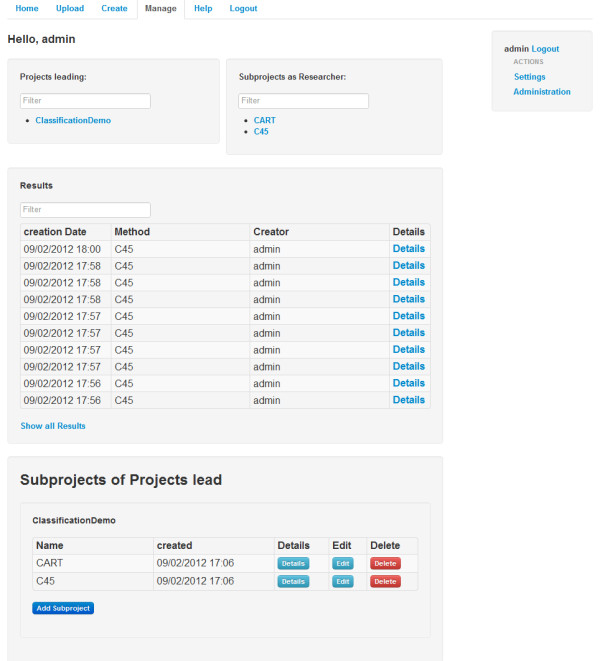
Project management screen.

The usage of the application depends greatly on the active user role, but as the general purpose of the software is the generation of relevant research data, this section will show some functionality from a researchers point of view. As mentioned above, a researcher can, if appointed as a project leader, administrate a project. This allows the user to create, edit and delete subprojects, share the project to users and assign other researchers to subprojects using the project management panel (see Figure 2). For every list or table within the framework, there is a JavaScript filtering option, to reduce clutter and improve efficiency while navigating through hierarchies.

**Figure 3:**
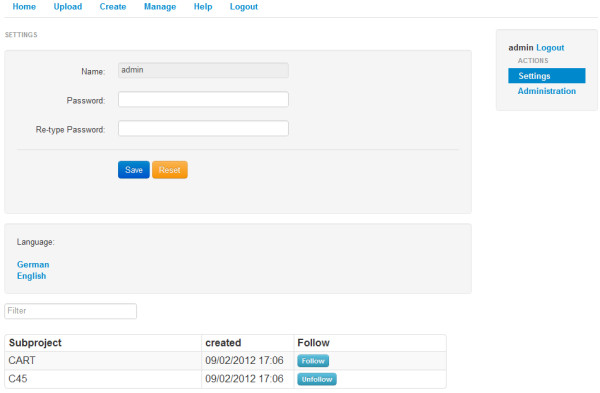
User settings.

Every user can change their own password, the language of the application and choose which content to follow, from the subprojects shared within the framework using the Settings-area (see Figure 3). Subprojects and their results can be shared to either the whole platform or to singular users. Once this content has been shared with a user, that user can select it from the Settings-panel and choose to follow it, the user can also view all results created within the shared subproject as well as comment on the visible subprojects and results.

**Figure 4:**
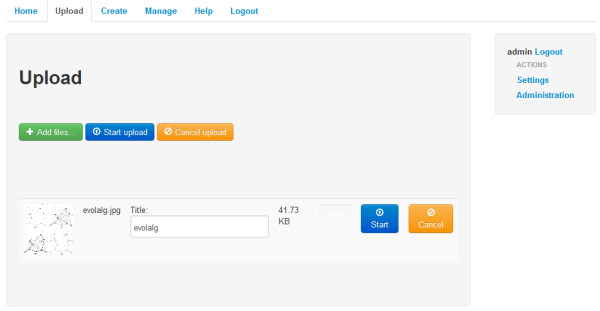
File upload screen.

Researchers can commit input data to the platform using the data upload feature (see Figure 4), consisting of a very user friendly jQuery widget, enabling even inexperienced users to just select files from their harddisk and upload them to the application. The widget also supports multiple parallel file uploads simultaneoously. The uploaded data can also be managed by a researcher within the management area.

**Figure 5:**
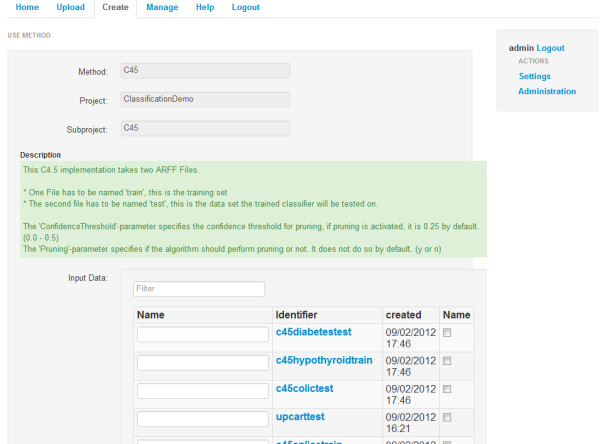
Result creation screen.

The most important process within KNODWAT is the generation of results, where a researcher chooses a project, a subproject and an algorithm, before proceeding to the result creation interface, where the chosen algorithm is documented and all the relevant parameters as well as input data can be specified. Once the result generation has been started, the algorithm is executed. When the method finishes successfully, the user receives an event notification. The created result can be viewed within the subproject detail page. The view can vary depending on the algorithm used, but usually consists of the name of the subproject, the used algorithm and input data, the comment section and algorithm specific result data such as visualizations and statistics about the result (example see Figure 5).

### Case study

In order to test KNODWAT with regard to its usability, stability, usefulness and the correctness of the two implemented methods, a small study was performed. In this study, the two implemented algorithms, CART and C4.5 were tested on two different data sets provided by the UCI [40]. The algorithms were trained using three different training set sizes (30%, 50% and 70%), with and without pruning, so that all in all there were 6 classifiers trained per method and data set. The results of this study are presented in the following section.

#### Test data

The test data was acquired from the UCI - University of California, Irvine machine learning data sets [40]. The two data sets used in this study were the Breast Cancer data set and the Hepatitis data set. Both have their origin in the medical domain. Example data rows for each set are: 

•Breast Cancer:

^′^50−59^′^,^′^*ge*40^′^,^′^15−19^′^,^′^0−2^′^,^′^*yes*^′^,^′^2^′^,^′^*left*^′^,^′^*central*^′^,^′^*yes*^′^,^′^*no*−*recurrence*−*events*^′^

^′^30−39^′^,^′^*premeno*^′^,^′^30−34^′^,^′^9−11^′^,^′^*no*^′^,^′^2^′^,^′^*right*^′^,^′^*left*_*u*_*p*^′^,^′^*yes*^′^,^′^*recurrence*−*events*^′^

•Hepatitis:

30, female, no, no, no, no, no, yes, no, no, no, no, no, 0.7, 100, 31, 4, 100, no, LIVE

59, female, no, no, yes, yes, no, yes, yes, yes, yes, no, no, 1.5, 107, 157, 3.6, 38, yes, DIE

#### Analysis

**Figure 6:**
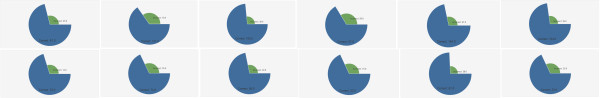
Results of all C45 classifiers.

**Figure 7:**
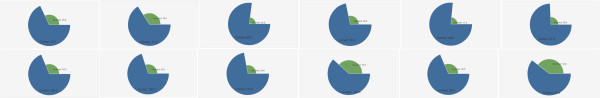
Results of all CART classifiers.

The result images in Figures 6 and 7, were taken directly out of the KNODWAT result detail views. They represent a graph showing the fraction of correctly and incorrectly classified instances for the different classifiers.

Obviously, the two data sets, each containing merely 300 to 500 data sets and only 6 different versions of the two algorithms to be tested on the data are not highly representative in a context of actually gaining useful knowledge and insights from the data. This was, however, not the goal of the study, which was to evaluate the functionality and framework that the KNODWAT application provides to conduct such studies. However, the results can be interpreted and compared, which can yield useful information on the usage and application of algorithms.

**Figure 8:**
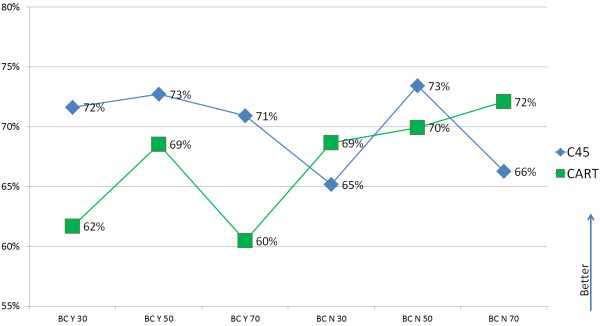
Comparison between CART and C4.5 on the breast cancer data set.

In Figure 8, the two algorithms, CART and C4.5 and the results generated from their application to the Breast Cancer data set are displayed. The graph shows, that on the whole, the C4.5 algorithm outperformed CART.

**Figure 9:**
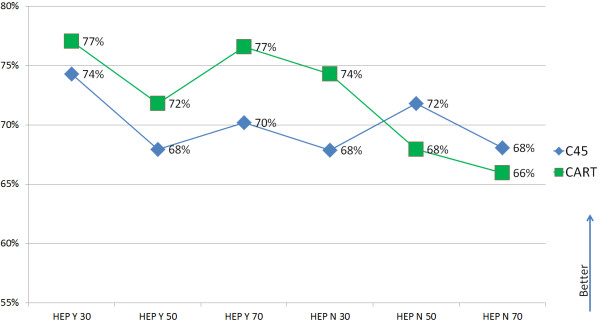
Comparison between CART and C4.5 on the hepatitis data set.

Figure 9 shows the comparison of CART and C4.5 on the Hepatitis data set, where the performance of the two algorithms is closer than on the Breast Cancer data set, and in this case, CART generated the best classifiers.

On the whole, the no-free-lunch theorem has been demonstrated in this small study as well, with each algorithm beating the other one on one of the data sets. Even in the case of these small data sets and a very limited range of different configurations using only two parameters, some fairly interesting results were generated by the use of the KNODWAT application. The program behaved as expected and made it very easy to conduct this study. With regard to the result, the multi-file upload, different subprojects for the two studies and the intuitive user interface, creation and viewing were the most impacting factors throughout the experience.

## Conclusions

This article introduced KNODWAT (Knowledge Discovery With Advanced Techniques), a framework for testing knowledge discovery methods with a focus on making it easy for developers to add new functionality to the existing system. KNODWAT is a web-based application created for researchers with a graphical user interface designed towards usability and easy access. Social features such as content sharing and the ability to express an opinion within the system as well as collaboration possibilities within projects, makes KNODWAT a modern environment for research groups. However, the application can not be extended without at least one expert who has programming experience and the skills to implement a certain knowledge discovery technique. The decision to create KNODWAT as a web-based application has both advantages and disadvantages. On the one hand, many users will have an easier time getting started with a web-based system due to the experience they have already gained with other systems of the kind, such as social networks or other prominent web sites. Web-based applications also have the advantage of being very connectable to external services and inherently create connections between users and their generated content. On the other hand, however, there may be limitations given methods with high computational complexity or very specific and expensive graphical representations of results, as they can be harder to implement in a web-based application, than in a native client. Nonetheless, with the trend of mobile devices becoming more and more capable and providing improved user interaction features, it was very important to make KNODWAT available for as many platforms as possible, which is definitely a strength of applications developed for the web.

On the whole, the idea of a globally connected research platform, making knowledge and the methods used to acquire it available to everyone, is very intriguing and KNODWAT is a small step in that direction.

## Availability and requirements

**Project name**: KNODWAT

**Project home page**: https://code.google.com/p/knodwat/

**Operating system(s)**: Platform independent

**Programming language**: Java

**Other requirements**: Java 1.6 or higher

**License**: Apache License 2.0

**Any restrictions to use by non-academics**: No

## Competing interests

We declare that we have not any competing interests.
